# Tceal5 and Tceal7 Function in C2C12 Myogenic Differentiation via Exosomes in Fetal Bovine Serum

**DOI:** 10.3390/ijms23042036

**Published:** 2022-02-12

**Authors:** Aika Sawada, Takuya Yamamoto, Takahiko Sato

**Affiliations:** 1 Faculty of Medical Sciences, Fujita Health University, Toyoake 470-1192, Japan; 41016052@fujita-hu.ac.jp; 2 Faculty of Medicine, Department of Anatomy, Fujita Health University, Toyoake 470-1192, Japan; 3 Center for iPS Cell Research and Application, Kyoto University, Kyoto 606-8507, Japan; takuya@cira.kyoto-u.ac.jp; 4 Institute for the Advanced Study of Human Biology (WPI-ASHBi), Kyoto University, Kyoto 606-8501, Japan; 5 Medical-Risk Avoidance Based on iPS Cells Team, RIKEN Center for Advanced Intelligence Project (AIP), Kyoto 606-8507, Japan; 6 International Center for Cell and Gene Therapy, Fujita Health University, Toyoake 470-1192, Japan

**Keywords:** myogenic differentiation, exosome, serum, Tceal5, Tceal7

## Abstract

The proliferation and differentiation of skeletal muscle cells are usually controlled by serum components. Myogenic differentiation is induced by a reduction of serum components in vitro. It has been recently reported that serum contains not only various growth factors with specific actions on the proliferation and differentiation of myogenic cells, but also exogenous exosomes, the function of which is poorly understood in myogenesis. We have found that exosomes in fetal bovine serum are capable of exerting an inhibitive effect on the differentiation of C2C12 myogenic cells in vitro. In this process of inhibition, the downregulation of *Tceal5* and *Tceal7* genes was observed. Expression of these genes is specifically increased in direct proportion to myogenic differentiation. Loss- or gain- of function studies with Tceal5 and Tceal7 indicated that they have the potential to regulate myogenic differentiation via exosomes in fetal bovine serum.

## 1. Introduction

Growth arrest and terminal differentiation of skeletal muscle cells to postmitotic myotubes occur when the level of serum is decreased in the cell-culture medium. Thus, in vitro studies with myogenic cells indicate that myogenic differentiation is controlled by serum depletion [1]. Myogenic differentiation in cell culture is usually inhibited by placing myoblasts into a mitogen-rich medium, in contrast to a mitogen-poor medium that promotes differentiation. These effects are due to several growth factors such as insulin-like growth factors, fibroblast growth factors, transforming growth factor-beta, platelet-derived growth factors, and so on [2,3,4,5,6].

It has recently been reported that serum contains not only various hormones and growth factors with specific actions on the proliferation and differentiation of myogenic cells, but also exogenous exosomes that are extracellular membrane micro-vesicles derived from many different types of cells and released into body fluids including blood [7,8]. Exosomes originate from endosome and carry a complex cargo of proteins, lipid, and nucleic acids, including DNA, mRNAs, and non-coding RNAs, functioning in intercellular communication [9]. Skeletal muscle cells also release extracellular micro-vesicles into the extracellular positions which represent new paracrine or endocrine signals to communicate to other organs [10,11]. However, little is known about the function of exosomes derived from non-muscle tissues to myogenic cells. It is important to investigate whether cultures of C2C12 myogenic cells could receive exosomes as target cells. Hence, we have analyzed the effect of micro-vesicles in serum to C2C12 cell cultures.

In this paper, we report that exosomes in fetal bovine serum are capable of exerting an inhibitive effect on the differentiation of C2C12 myogenic cells in vitro, and *Tceal5* and *Tceal7* genes, encoding members of the transcription elongation factor A (Sll)-like gene family [12,13], are expressed during myogenic differentiation and are dynamically regulated by exosomes in serum. It has been reported that members of this family, with a zinc finger-like motif, are ubiquitously expressed in normal tissues (Tceal1 or Tceal4), and function as nuclear phosphoproteins to modulate the transcription in a promoter context-dependent manner. It exerts its effects via protein–protein interactions with other transcriptional regulators. Multiple family members are located on the X chromosome [12].

Our studies show that exosomes function as upstream regulators of *Tceal5* and *Tceal7* gene expression and indicate the role of both genes during muscle differentiation. These results define an exosome–Tceal cascade, which is important in the regulation of muscle differentiation.

## 2. Results

### 2.1. Myogenic Cell Growth without Exosomes in Serum

**Figure 1:**
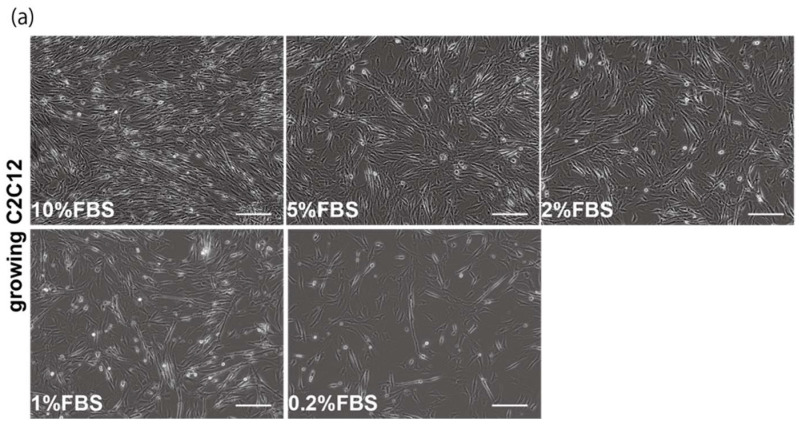

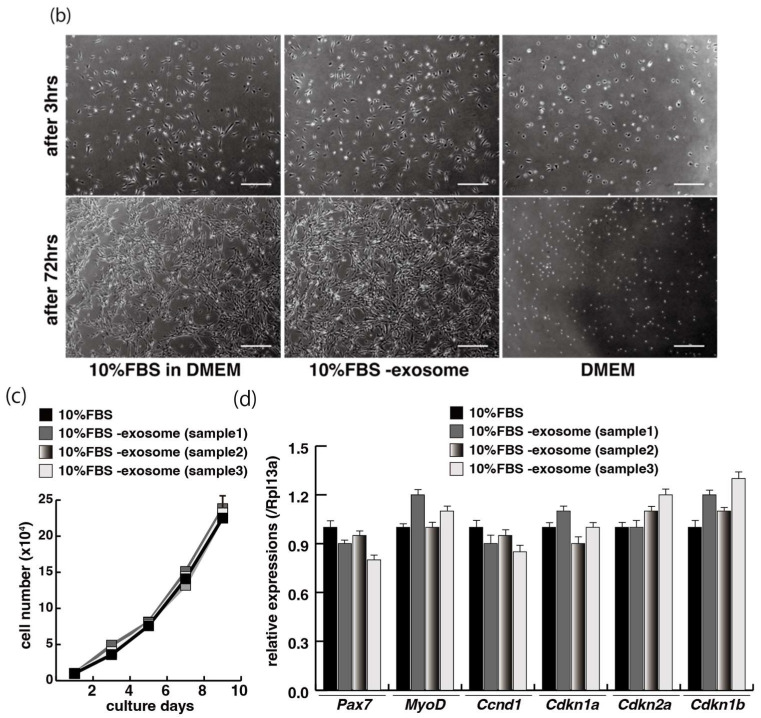
Exosome-depleted serum does not affect myogenic cell growth. (**a**) Cultured C2C12 cells with different dilutions of fetal bovine serum (FBS); (**b**) morphology of growing C2C12 cells with or without exosome in FBS (10%FBS in DMEM or 10%FBS−exosome) and without serum (DMEM); (**c**) C2C12 cells grow in the condition with or without exosomes (sample1–3); (**d**) relative mRNA expression of myogenic stem cell (*Pax7*), myogenic commitment to differentiation (*MyoD*), and cell cycle (*Ccnd1*, *Cdkn1a*, *cdkn2a*, *Cdkn1b*) markers in C2C12 cells cultured with or without exosomes in FBS. All Scale bar = 100 μm. Data represent means ± SEM (*n* = 3). The statistical analysis was performed by Mann–Whitney U test.

The proliferation and differentiation of skeletal muscle cells in culture are usually controlled by serum components. To define the appropriate serum condition for the growth of C2C12 myoblast cells, we grew these cells in culture medium supplemented with different concentrations of serum. Cells cultured in 10% of FBS (fetal bovine serum) in DMEM reached confluence within a few days (Figure 1a, upper left panel), not observed with less than 10% of FBS in DMEM, as mentioned in many protocols (Figure 1a). It has been reported that many growth factors in serum can stimulate myogenic proliferation and differentiation, and recently exosomes, as well as growth factors, have been identified in serum. To evaluate the effect of exosomes in serum on myogenic cell growth, we prepared exosome-depleted serum, which is available as a commercial product, for proliferating C2C12 cells (Figure 1b, middle panels). There was no apparent morphological difference with or without exosomes in these cultures (Figure 1b, left and middle panels). C2C12 cells did not grow in non-serum conditions although the same number of cells were initially plated (Figure 1b, right panels). Under conditions where exosomes were or were not present in the serum, the number of growing cells was similar, and the level of transcripts for markers of myogenic stem cells, myogenic commitment to differentiation, and the cell cycle showed no difference (Figure 1c,d).

### 2.2. Myogenic Differentiation with Exosomes from Serum

**Figure 2:**
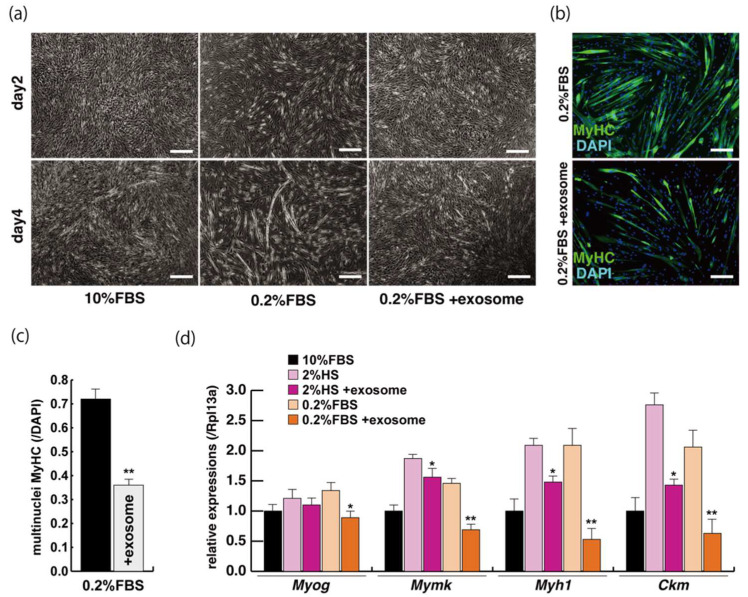
Addition of exosomes derived from serum affects myogenic differentiation. (**a**) Approximately 80% of semi-confluent C2C12 cells with 10% or 0.2% of fetal bovine serum (FBS) with or without additional exosomes; (**b**) differentiated C2C12 cells (day4) were immunostained with anti-MF20 (MyHC, green). All nuclei were stained with 4′6-diamidino-2-phenylindole (DAPI, blue); (**c**) the ratio of DAPI-positive multinuclei staining present in single MyHC-positive myofibers with or without additional exosomes; (**d**) relative expression of myogenic differentiation (*Myog*), fusion (*Mymk*), and differentiated markers (*Myh1*, *Ckm*) in C2C12 cells cultured in FBS with or without additional exosomes. All Scale bar = 100 μm. Data represent means ± SEM (*n* = 3). The statistical analysis was performed with Mann–Whitney U test. * *p* < 0.05, ** *p* < 0.01.

The incubation of C2C12 cells in low-serum conditions can promote myogenic differentiation and we next investigated whether this is impacted by exogenous exosomes from serum impact. Cells cultured in 10% of FBS in DMEM reached semi-confluence around 80% within a few days (Figure 2a, upper panels), and elongated myotubes appeared on the second day after switching to the low serum condition of 0.2% of FBS in DMEM (Figure 2a, lower middle panel). When exosomes isolated from FBS (Supplementary Figure S1) were added to the low-serum medium, negative morphological effects on myogenic differentiation were observed (Figure 2a, lower right panel). In this differentiated phase, myosin heavy chain (MyHC) staining, as a myogenic differentiation marker, was present in a lower percentage of total cells in the presence of additional exosomes (Figure 2b,c). Lower level of transcripts of the myogenic differentiation factor (myogenin: *Myog*), fusion (myomaker: *Mymk*), and maturation markers (myosin heavy polypeptide1: *Myh1,* Creatine kinase: *Ckm*) were also observed under these conditions (Figure 2d, Supplementary Figure S2). These data suggest that exosomes in fetal bovine serum function to myogenic differentiation, not to cell growth.

### 2.3. Tceal5 and Tceal7 in Differentiated C2C12 Cells

**Figure 3:**
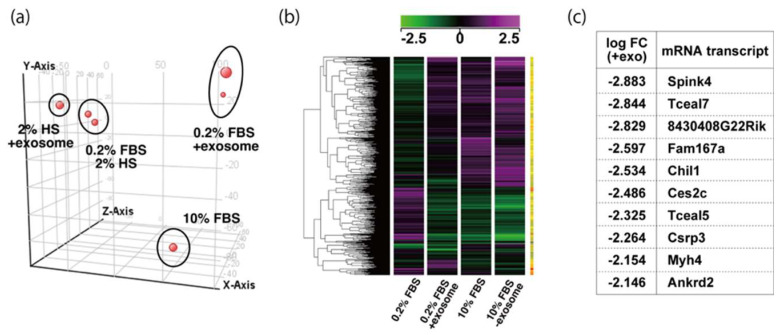

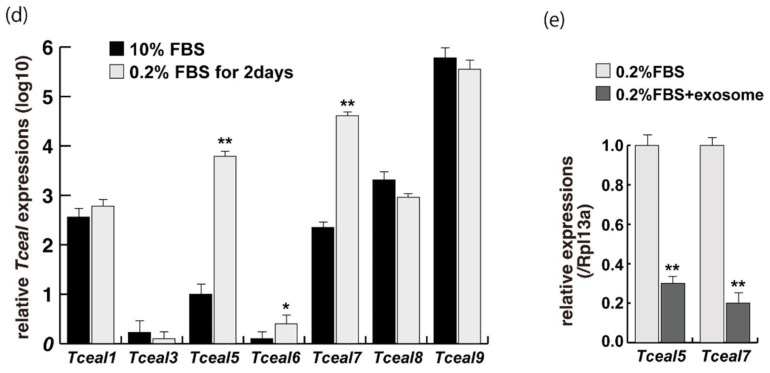
Identification of downregulated genes in differentiating myogenic cells cultured in the presence of added exosomes. (**a**) Principal component analysis, (**b**) heatmap of NGS data, illustrating differentially expressed messenger RNAs between 0.2% of FBS differentiating medium (0.2% FBS), 0.2% of FBS supplemented with exosomes (0.2% FBS + exosome), 10% of FBS (10% FBS), and 10% of exosome-depleted FBS (10% FBS −exosome); (**c**) TOP10 downregulated genes in differentiating C2C12 cells cultured in the presence of additional exosomes; (**d**) transcriptional changes in the *Tceal* family of genes in growing (10% FBS) and differentiating (0.2% FBS) C2C12 cells; (**e**) relative RT-qPCR of *Tceal5* and *Tceal7* transcripts in differentiating muscle cells with or without additional exosomes. Data represent means ± SEM (*n* = 3). The statistical analysis was performed by Mann–Whitney U test. * *p* < 0.05, ** *p* < 0.01.

To examine the effect of exosome in serum on myogenic differentiation, we performed next-generation sequencing analyses (NGS) with C2C12 cells in growing conditions with 10% of FBS, or in differentiating conditions with 0.2% of FBS or 2% of horse serum with or without added exosomes (Figure 3a,b, Supplementary Table S1). Among downregulated mRNAs expressed in differentiating C2C12 cells with the addition of exosomes, the expression levels of *Tceal5* and *Tceal7* were significantly lower in both NGS and RT-qPCR analyses (Figure 3c–e). These data suggest that *Tceal5* and *Tceal7* are upregulated in differentiating myogenic cells and that exogenous exosomes in fetal bovine serum downregulated less than half of these transcripts.

### 2.4. Attenuated Tceal5 and Tceal7 Expression Effects to Myogenic Differentiation

**Figure 4:**
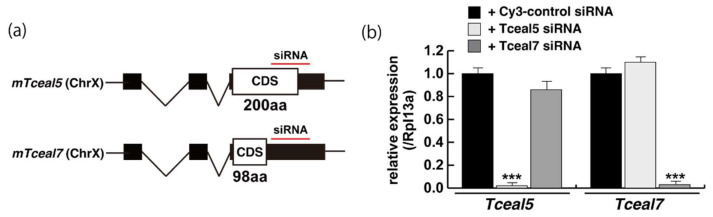

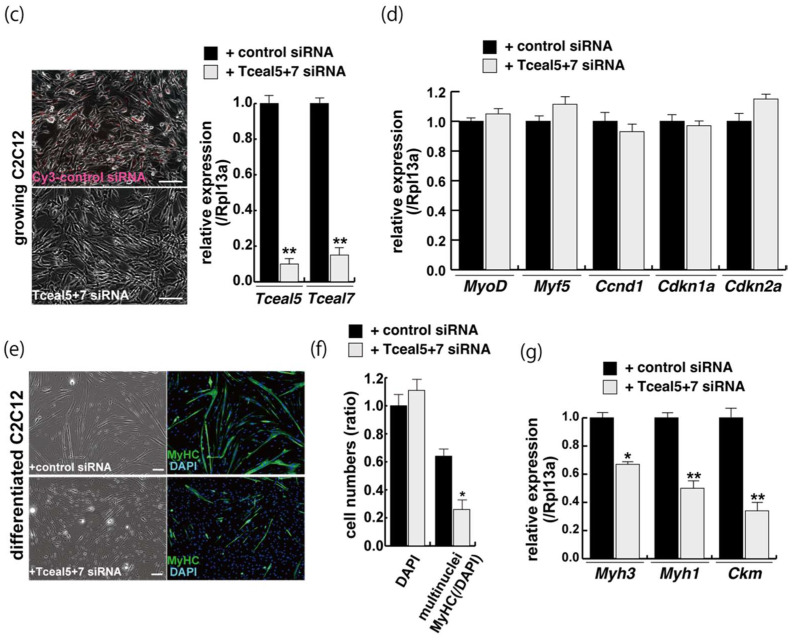
Downregulation of Tceal5/7 reduced myogenic differentiation. (**a**) Schematic representation of mouse Tceal5 (mTceal5, coding sequence; 200 amino acids) and Tceal7 (mTceal7, coding sequence; 98 amino acids) on chromosome X (ChrX); (**b**) RT-qPCR analyses after siRNA transfection into C2C12 cells. Each siRNA represses transcripts from the targeted gene specifically; (**c**) morphological features with both Tceal5 and Tceal7 siRNA treatment in growing C2C12 cells. Cy3-labelled siRNA was used as a control (left). RT-qPCR analyses after both siRNAs transfection into C2C12 cells (right). (**d**) Relative RT-qPCR analyses of myogenic determination (MyoD, Myf5) and cell cycle genes (Ccnd1, Cdkn1a, Cdkn2a) in growing C2C12 cells with or without both siRNAs; (**e**) differentiated C2C12 cells with siRNAs were immunostained with anti-MF20 (MyHC, green). All nuclei were stained with 4′6-diamidino-2-phenylindole (DAPI, blue); (**f**) relative cell numbers of differentiated C2C12 cells with or without Tceal5/7 siRNAs, counted with anti-MyHC antibody and DAPI in (**e**); (**g**) relative RT-qPCR of mature myogenic transcripts (Myh1, Myh3, Ckm) in differentiating muscle cells with or without Tceal5/7 siRNAs; All Scale bar = 100 μm. Data represent means ± SEM (*n* = 3). The statistical analysis was performed by Mann–Whitney U test. * *p* < 0.05, ** *p* < 0.01, *** *p* < 0.001.

We next examined whether the knockdown of *Tceal5/7* mRNAs with small interfering RNAs (siRNAs) affected differentiating C2C12 cells in a similar manner to the addition of exosomes. The locations of target positions of siRNAs in the coding sequence (CDS) and 3′UTR of *Tceal5* and *Tceal7* genes are indicated in Figure 4a. Treatment with each siRNA showed the repression of the targeted transcripts specifically (Figure 4b). C2C12 cells grown with these siRNAs were morphologically normal (Figure 4c), and their level of transcripts related to myogenesis, or the cell cycle was almost identical to that of control C2C12 cells (Figure 4d). 

Downregulation of single Tceal5 or Tceal7 did not show significant reduction of myogenic differentiation (Supplementary Figure S3). In contrast, the treatment with both Tceal5 and Tceal7 siRNAs had an impact on differentiated myofibers in C2C12 cultures, shown as lower staining efficiency with MyHC antibodies and a reduction in transcripts of myogenic maturation markers compared to non-treated cells (Figure 4e,f). These results suggest that the attenuation of *Tceal5* and *Tceal7* gene expression had an effect on myogenic differentiation.

### 2.5. Increased Expression of Tceal5 and Tceal7 Rescues Myogenic Differentiation from the Negative Effects of Exosomes

**Figure 5:**
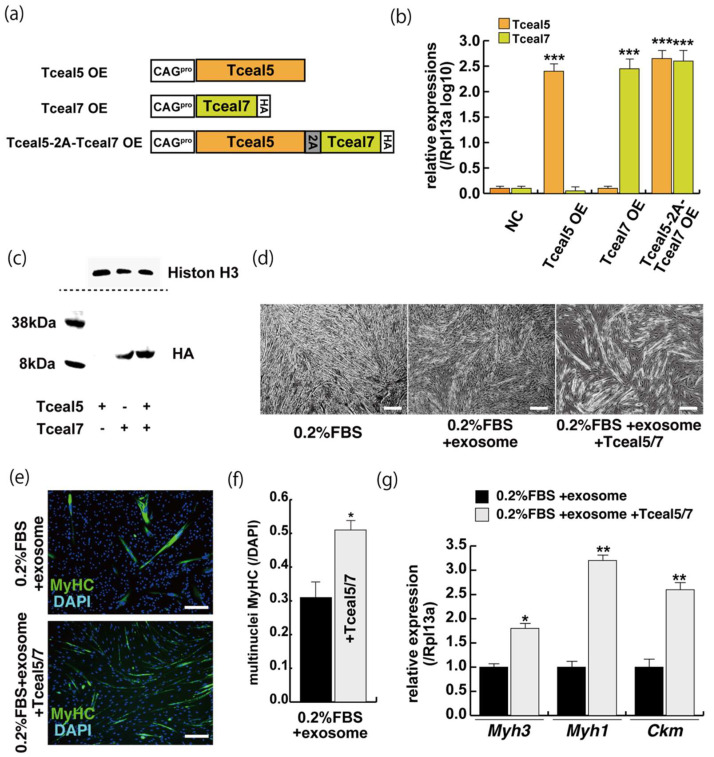
The upregulation of Tceal5/7 rescues differentiating myogenic cells repressed by exosome. (**a**) Representation of DNA constructs for overexpression of mouse Tceal5 (Tceal5 OE), HA-tagged Tceal7 (Tceal7 OE), and both genes (Tceal5-2A-Tceal7 OE); (**b**) RT-qPCR analyses with Tceal5 and/or Tceal7 overexpressing in C2C12 cells; (**c**) Western blot analyses with Tceal5 and/or Tceal7 overexpressing 293 cells. Histone H3 was used as a loading control; (**d**) morphological changes with both Tceal5 and Tceal7 overexpression in differentiated C2C12 cultures in the presence of additional exosomes; (**e**) differentiated C2C12 cells treated with additional exosomes and Tceal5/7 overexpressions, were immunostained with anti-MF20 (MyHC, green). All nuclei were stained with 4′6-diamidino-2-phenylindole (DAPI, blue); (**f**) The ratio of DAPI-positive multinuclei staining present in single MyHC-positive myofibers with additional exosomes and Tceal5/7 overexpression; (**g**) relative RT-qPCR of mature myogenic transcripts (Myh1, Myh3, Ckm) in differentiating muscle cells with exosomes and Tceal5/7; All Scale bar = 100 μm. Data represent means ± SEM (*n* = 3). The statistical analysis was performed by Mann–Whitney U test. * *p* < 0.05, ** *p* < 0.01, *** *p* < 0.001.

These studies suggest that *Tceal5* and *Tceal7* genes are downregulated by exosomes. To investigate further their functional role in myogenic differentiation, we examined the effects of Tceal5/7 overexpression, using vectors producing, Tceal5, HA-tagged Tceal7, and 2A peptide-based bicistronic Tceal5 and Tceal7 proteins, driven by a CAG promoter (Figure 5a). Tceal5 and Tceal7 overexpression were confirmed by RT-qPCR (Figure 5b), cleaved Tceal7-HA-tag protein was detected by Western blot analyses (Figure 5c) and Tceal7-HA was localized in the nucleus by immunostaining with anti-HA antibody (Supplementary Figure S5). To further investigate their functional roles in myogenic differentiation, C2C12 cells were seeded in growth condition with or without additional expression of Tceal5/7 factors and then exposed to myogenic differentiating condition with or without additional exosomes as shown in Figure 2a. Differentiating medium with added exosomes suppressed myogenic differentiation, however this effect was partially rescued by Tceal5/7-overexpression as shown by morphological elongation of myogenic cells and MyHC staining (Figure 5d–f). Transcripts of differentiated myogenic markers were upregulated in cells grown under the condition of Tceal5/7 overexpression (Figure 5g). The upregulation of single Tceal5 or Tceal7 did not rescue the phenotype that exosomes repressed myogenic differentiation (Supplementary Figure S4). These results support the hypothesis that exosomes in fetal bovine serum downregulate the expression of Tceal5 and Tceal7, which are necessary for myogenic differentiation. 

## 3. Discussions

Proliferation and terminal differentiation of myogenic cells are usually mutually exclusive. When growing myogenic cells are induced to differentiate by being placed in a low-serum condition, they withdraw from the proliferative cell cycle because of the mitogen-poor condition. We initially tested whether exosomes, reported as novel serum components, might act as regulators of cell growth and differentiation in myogenesis. To isolate intact exosomes from FBS, we used commercial kits involving a simple and quick precipitation method, allowing them to be collected by a short, low-speed centrifugation easily applicable in most research laboratories [14]. In our approach, FBS-derived exosomes, from two different batch preparations had a significantly negative effect on myogenic differentiation, although exosome-depleted serum did not have any effect to cell growth of C2C12 cells. 

The results of screening differentiated C2C12 cells, after administration of exosomes in the culture medium, showed that myogenic differentiation was compromised, with changes in gene expression. We particularly focused on Tceal5 and Tceal7 which were highly downregulated in the presence of exosomes in differentiating C2C12 cells. The Tceal, transcription elongation factor A (SII)-like family is characterized by a TFA (transcription elongation factor A) domain. This gene family includes nine members in the human genome and seven members in the mouse genome. Tceal1, the prototypical member of this family, participates in RSV (Rous sarcoma virus) long terminal repeat repression [15]. Tceal7 has been associated with tumor suppression and shown to be negatively regulated by miR-182 [16,17]. No function for Tceal5 has been reported in myogenesis, however Tceal7 was identified as a gene expressed in muscle cells and directly controlled by myogenic regulatory factors and androgen receptor [18,19]. Its overexpression negatively affected proliferation and promoted myogenic differentiation [18]. We now show that both Tceal5 and Tceal7 affect the progression of myogenic differentiation ant that their expression in differentiating muscle cells is negatively controlled by exosomes present in fetal bovine serum. The next important challenges are to identify the molecules, including microRNAs, present in exosomes derived from fetal bovine serum that regulate the expression of these factors and to determine by what mechanism these factors directly or indirectly affect myogenic differentiation, and analyze exosomes derived from murine and human sera.

Skeletal muscle tissues have a remarkable capacity to repair, however these processes, including myogenic differentiation, fail and ultimately result in the collapse of the muscles in muscular diseases such as Duchenne muscular dystrophy. The understanding of myogenic regulatory cascades is important to elucidate the molecular mechanisms of myogenic differentiation and regeneration with a view to developing innovative therapies for these patients. The presence of Tceal7 has been reported in regenerating skeletal muscle after cardiotoxin injury [18], and it will be important to follow up on this observation, as well as on the possible presence and role of Tceal5 during muscle regeneration. Our results now also add the dimension of exosome regulation of these understudied factors in the context of muscle differentiation and repair of injured muscle.

## 4. Materials and Methods

### 4.1. Isolation of Exosomes from FBS and C2C12 Myogenic Cell Cultures

Exosome-depleted fetal bovine serum (Exosome-Depleted FBS, ThermoFisher Scientfic, Waltham, MA, USA) and normal FBS (lot No.1556664, 1632535, ThermoFisher Scientific, Waltham, MA, USA) were prepared. Exosome fractions from FBS were isolated using miRCURY Exosome Isolation Kit (Exicon, Vedbaek, Denmark), ExoQuick Exosome Precipitation Solution (System Biosciences, Palo Alto, CA, USA), and total Exosome Isolation from serum (Thermo Fisher Scientific) according to the manufacturer’s instructions.

C2C12 myogenic cells were cultured in DMEM containing 10% of FBS with or without exosomes. After a few days of cell culture at 70–80% of confluency, these cells were differentiated into myotubes in DMEM supplemented with 2% horse serum (Thermo Fisher Scientific) or 0.2% FBS with or without additional exosomes derived from FBS. For example, extracted exosomal fraction derived from 5 mL of FBS were resuspended into 1ml of elution buffer, and added to 25 mL, 50 mL, 100 mL, and 250 mL of 0.2% FBS in DMEM culture medium, nearly equals the number of exosomes in 20%, 10%, 5%, and 2.5% FBS, respectively. All experiments with exosomes (0.2% FBS or 2% HS + exosome) were performed as the number of exosomes involved in 10% FBS.

### 4.2. Next-Generation Sequencing and RT-qPCR Analyses

Total RNAs from cultured C2C12 or 293T cells were extracted using RNeasy Micro Kit (Qiagen, Hilden, Germany). The 500 ng of total RNAs were used as starting materials to generate RNA-seq libraries with the TruSeq Stranded mRNA LT sample prep kit (Illumina, San Diego, CA, USA). The obtained libraries were sequenced on a NextSeq500 (Illumina) as 75 bp single-end reads. After trimming adaptor sequences and low-quality bases with cutadapt-1.9.1 [20], the sequenced reads were mapped to the mouse reference genome (mm10) with TopHat2 v2.1.0 [21], and expression levels were calculated as FPKM using Cufflinks v2.2.1 [22]. FPKM values are imported into GeneSpring GX software version 14.9.1 (Agilent Technologies), and differentially expressed genes were identified by with log2FC > 1 or < −1. The raw data has been deposited as GSE193702.

For quantitative PCR analyses, synthesized cDNA was prepared using the SuperScript VILO kit (Invitrogen, Waltham, MA, USA) from mRNAs. All RT-qPCR reactions were carried out in triplicate using THUNDERBIRD SYBR qPCR Mix (Toyobo, Osaka, Japan), Thermal Cycler Dice Realtime System (Takara, Shiga, Japan) and normalized to the mRNA expression level of ribosomal protein L13A (Rpl13a) as a control. Primer sequences (5′ to 3′) are listed in Supplemental Table S2.

### 4.3. Immunofluorescence

Cultured cells were fixed with 4% paraformaldehyde (Nacalai, Kyoto, Japan) in PBS for 10 min at 4 °C. Fixed samples were incubated with anti-MyHC antibody (MF20, R&D Systems, Minneapolis, MN, USA; diluted 1/200), anti-HA (Cell Signaling Technology; diluted 1/200) in 5% of BlockingOne (Nacalai) overnight at 4 °C. After three washes with 0.1% of Tween20 in PBS, cells were incubated with Alexa488-conjugated secondary antibodies (Molecular Probes, Eugene, OR, USA; diluted 1/500). Cells were washed and mounted in SlowFade Diamond Antifade Mountant with DAPI (Molecular Probes). Images were collected and processed to change original fluorescent colors, and count cell numbers when appropriate on the software of BZX-710 (Keyence, Osaka, Japan). For quantitation of cultured cells, the numbers of dishes were analyzed from triplicate experiments.

### 4.4. Western Blot

The cells were lysed with radio-immunoprecipitation assay (RIPA) buffer containing protease inhibitor cocktail (Nacalai). Following centrifugation, the supernatant containing the total proteins was fractionated by sodium dodecyl sulfate (SDS)-poly-acrylamide gel electrophoresis (TEFCO, Hachioji, Tokyo, Japan). The separated proteins were transferred to polyvinylidene difluoride membranes (TEFCO), blocked with 5% of BlockingOne (Nacalai) for 30 min, and incubated with anti-HA (Cell Signaling Technology; diluted 1/1000), anti-HistonH3 (Cell Signaling Technology; diluted 1/1000), anti-CD9 (WAKO; diluted 1/500), and anti-CD81 (WAKO; diluted 1/500) primary antibodies overnight at 4 °C. The blots were probed with horseradish peroxidase-conjugated secondary antibodies (Molecular Probes; diluted 1/5000) and developed with luminal for enhanced chemiluminescence using Chemi-Lumi One Super (Nacalai). When probing for multiple targets, a single membrane was stripped with WB Stripping Solution (Nacalai) and re-probed with antibodies again.

### 4.5. Tceal Overexpression and siRNA Constructs

For overexpression in C2C12 or 293T cells, pCAGGS plasmid vector (RIKEN RDB08938) was used. *Tceal5* and *Tceal7* coding sequences were amplified by RT-PCR with KOD FX Neo DNA polymerase (Toyobo) and single strand cDNA synthesized by SuperScript4 Reverse Transcriptase (Invitrogen) from C2C12 cells. These cDNAs were subsequently subcloned into the pTA2 vector (Toyobo) to confirm the whole sequences, and then ligated into pCAGGS vector (Tceal5 OE, Tceal7 OE). The P2A sequence was amplified by synthesized oligos with P2A-nanolantern vector [23] and cloned into the position after the deleted stop codon of *Tceal5*, ligated into *Tceal7* CDS (Tceal5-2A-Tceal7 OE). Plasmid DNA was introduced into cells with ViaFect reagent following the manufacturer’s instruction (Promega, Madison, WI, USA).

For transfection with each 50 nM of MISSION esiRNAs targeted for mouse *Tceal5* (EMU092641, Sigma-Aldrich, St. Louis, MO, USA) or *Tceal7* (EMNC006071, Sigma-Aldrich, St. Louis, MO, USA), cells were incubated with Lipofectamine RNAiMAX (Invitrogen) according to the manufacturer’s protocols.

### 4.6. Statistics

We report the statistical data, including the results of at least three biological replicates. Statistical analyses were performed with StatPlus software (AnalystSoft, mac LE, Walnut, CA, USA) to determine significant differences with Mann—Whitney U test for two-sample comparison. *p*-values indicated on each figure as * *p* < 0.05, ** *p* < 0.01, and *** *p* < 0.001. All error bars are indicated as means ± SEM (*n* = 3).

## Data Availability

Not applicable.

## Supplementary Materials

The following supporting information can be downloaded at: https://www.mdpi.com/article/10.3390/ijms23042036/s1.
