# Supplementary material for: Tceal5 and Tceal7 Function in C2C12 Myogenic Differentiation via Exosomes in Fetal Bovine Serum
Source: Int J Mol Sci. 2022 Feb 12;23(4):2036. doi: 10.3390/ijms23042036 (PMC8877866; doi:10.3390/ijms23042036)
Supplement: Supplementary file 1 [file ijms-23-02036-s001.zip › ijms-1518644-supplementary.pdf]

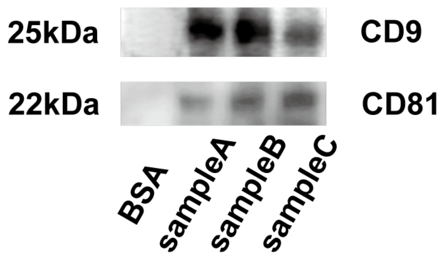

Supplementary Figure S1. Analysis of exosomes in fetal bovine serum by western blot. Western blot analysis of exosome markers CD9 and CD81 on 10μg of lysate using commercial exosome isolation kits. sample A; miRCURY Exosome Isolation Kit (Exicon), sample B; ExoQuick Exosome Precipitation Solution (System Biosciences), sample C; total Exosome Isolation from serum (Thermo Fisher Scientific). BSA was used as negative control.

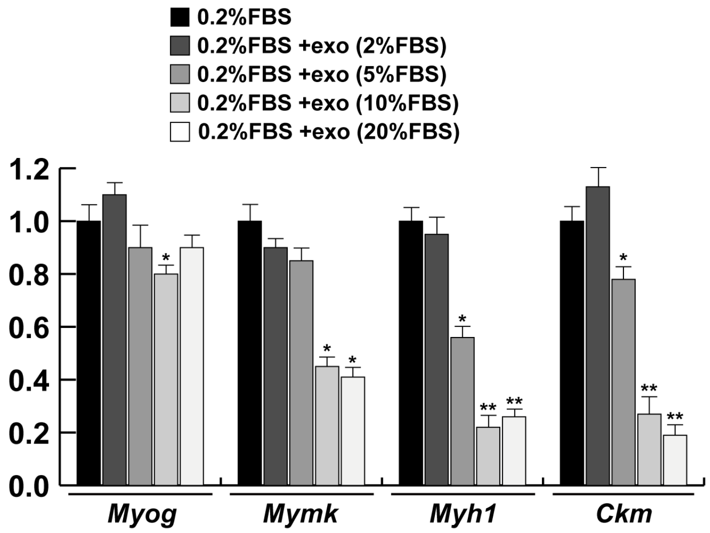

Supplementary Figure S2. Myogenic differentiation was inhibited in proportion as the total amounts of exosomes derived from fetal bovine serum. Relative expression of myogenic differentiation (Myog), fusion (Mymk), and differentiated markers (Myh1, Ckm) in C2C12 cells cultured in FBS with additional exosomes. Data represent means ± SEM (n = 3). The statistical analysis was performed with Mann-Whitney U test. *P<0.05, **P<0.01.

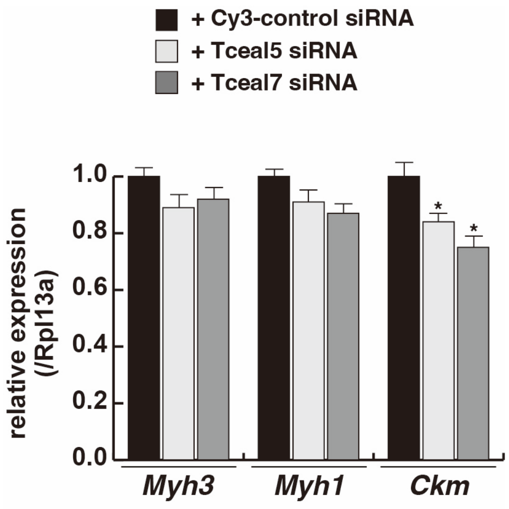

Supplementary Figure S3. Downregulation of single Tceal5 or Tceal7 does not show significant reduction of myogenic differentiation. Relative RT-qPCR of mature myogenic transcripts (Myh1, Myh3, Ckm) in differentiating C2C12 muscle cells with Tceal5 or Tceal7 siRNAs. Data represent means ± SEM (n = 3). The statistical analysis was performed by Mann-Whitney U test. *P<0.05.

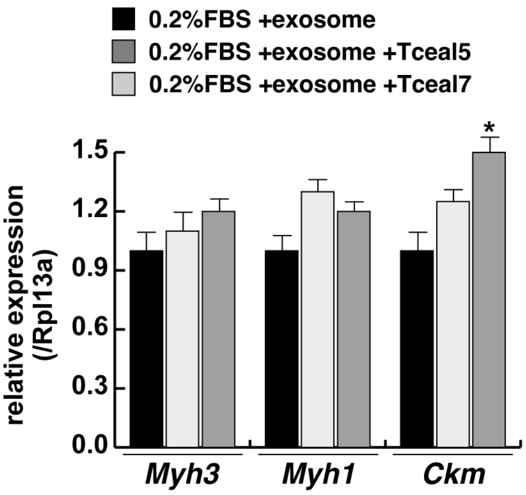

Supplementary Figure S4. The upregulation of single Tceal5 or Tceal7 does not rescue differentiating myogenic cells repressed by exosome. Relative RT-qPCR of mature myogenic transcripts (Myh1, Myh3, Ckm) in differentiating muscle cells with exosomes with the acceleration of Tceal5 or Tceal7. Data represent means ± SEM (n = 3). The statistical analysis was performed by Mann-Whitney U test. *P<0.05.

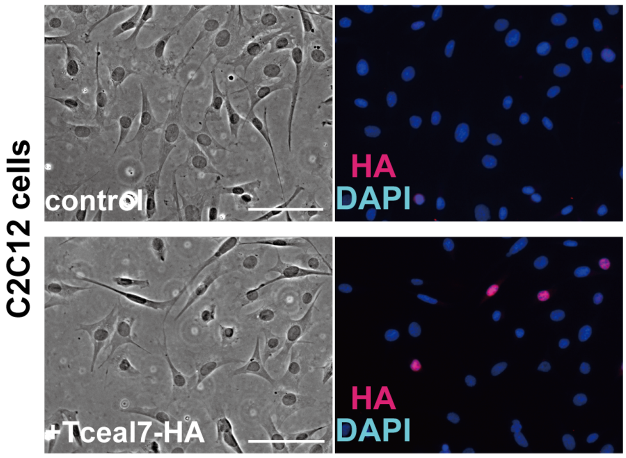

Supplementary Figure S5. C2C12 cells, transfected with the Tceal7-HA construct, were immunostained with anti-HA antibody (red, lower panels). All nuclei were stained with 4′6-diamidino-2-phenylindole (DAPI, blue). All Scale bar=100 μm.

Supplementary Table S1. NGS data of differentiating C2C12 cells with or without additional exosomes

tracking_id	Log FC (FBS+exo vs FBS)	FC (abs) (FBS+exo vs FBS)	Regulation after +exo
Spink4	-2.8830435	7.3770475	down
Tceal7	-2.8439162	7.179663	down
8430408G22Rik	-2.8286142	7.1039147	down
Fam167a	-2.5971773	6.0510154	down
Chil1	-2.534428	5.793471	down
Ces2c	-2.4858043	5.6014657	down
Tceal5	-2.3253999	5.012047	down
Csrp3	-2.2640836	4.803492	down
Myh4	-2.1543949	4.451819	down
Ankrd2	-2.1464772	4.4274535	down
Tigit	-2.0593433	4.1679654	down
Gm17455	-1.9875376	3.9655957	down
Hsd11b1	-1.9763135	3.934863	down
Actn2	-1.9734614	3.927092	down
Filip1	-1.9710255	3.920467	down
Bglap2	-1.970986	3.9203596	down
Murc	-1.9655819	3.905702	down
Tcap	-1.9441966	3.8482342	down
Myo18b	-1.9232471	3.7927575	down
Kcnk13	-1.9230478	3.7922335	down
Mttp	-1.9056093	3.746671	down
Lmod2	-1.8855109	3.6948376	down
Unc13d	-1.885422	3.6946099	down
Myh8	-1.8820603	3.6860108	down
Macc1	-1.8649402	3.6425283	down
Cox6a2	-1.8619585	3.6350079	down
Ctxn3	-1.8559158	3.6198146	down
R3hdml	-1.8325586	3.5616817	down
Bex1	-1.8247089	3.5423553	down
Smpx	-1.8159356	3.520879	down
Pi15	-1.7996709	3.481408	down
Klhl13	-1.7968483	3.4746034	down
Sox11	-1.7929769	3.4652917	down
Adprhl1	-1.7919044	3.4627168	down
Txlnb	-1.7543485	3.3737392	down
Tnni3	-1.7404402	3.341371	down
Pkhd1	-1.7400914	3.3405633	down
Dclk1	-1.7376461	3.334906	down
Krt80	-1.7364535	3.3321505	down
Myl6b	-1.7257304	3.3074753	down
Rap1gap2	-1.7239115	3.303308	down
Cacng6	-1.7231612	3.3015907	down
Shroom3	-1.7133762	3.2792733	down
Otog	-1.7122349	3.2766802	down
Sohlh2	-1.7102388	3.2721498	down
Tnnc1	-1.6687107	3.1793034	down
Tmem182	-1.6661353	3.173633	down
Rab44	-1.653092	3.1450698	down
Colq	-1.6511413	3.14082	down
Susd5	-1.6487709	3.135664	down
Lamc2	-1.6476228	3.1331694	down
Lman1l	-1.6359881	3.1080034	down
Lmod3	-1.6355829	3.1071308	down
Mef2c	-1.6146166	3.062302	down
Dusp9	-1.6139369	3.0608597	down
Adm2	-1.6138053	3.0605805	down

Igf2	-1.6130953	3.0590746	down
Apobec2	-1.6047347	3.041398	down
Dusp13	-1.6015637	3.0347207	down
BC051019	-1.6008112	3.0331383	down
Hbegf	-1.5855716	3.001267	down
Fn3k	-1.5840032	2.9980059	down
Armcx4	-1.5816432	2.9931056	down
Myh1	-1.5719624	2.9730885	down
Mylpf	-1.5684013	2.965759	down
Adora1	-1.5661573	2.9611495	down
Map3k7cl	-1.5625427	2.9537396	down
Nrxn3	-1.5597023	2.94793	down
Pstpip1	-1.5596988	2.947923	down
Atp1a2	-1.5585582	2.9455934	down
Cdkn1c	-1.5546379	2.9376	down
Tnnt3	-1.5469465	2.9219804	down
Hspb3	-1.5452726	2.918592	down
Itgb1bp2	-1.5435376	2.9150844	down
Mybph	-1.5326843	2.8932366	down
Dhrs7c	-1.5199125	2.8677366	down
Tekt5	-1.5134989	2.8550162	down
Fgf21	-1.5017242	2.8318095	down
Slc5a5	-1.498967	2.8264027	down
Adamtsl2	-1.4985247	2.8255363	down
Acox2	-1.4912835	2.81139	down
Synpo2l	-1.4874134	2.8038583	down
Tnni1	-1.4857903	2.8007054	down
Myl4	-1.4821348	2.7936182	down
Tnni2	-1.4819593	2.7932785	down
Cfap52	-1.4753369	2.7804856	down
C1qtnf3	-1.4667401	2.7639666	down
Ccdc162	-1.4659598	2.762472	down
Dbh	-1.4640357	2.7587903	down
Myl1	-1.4581423	2.7475433	down
Prrg4	-1.4381465	2.7097251	down
Hspb2	-1.437468	2.708451	down
Trib3	-1.4341455	2.7022207	down
Cav3	-1.4339294	2.701816	down
Ddc	-1.4236405	2.6826158	down
Slc2a6	-1.4216533	2.6789234	down
Myom2	-1.4195905	2.6750956	down
Ankrd1	-1.4150801	2.6667454	down
Xirp1	-1.4088564	2.655266	down
Plekha7	-1.4066708	2.6512465	down
Anxa8	-1.4038146	2.6460028	down
Asns	-1.4012632	2.6413276	down
Rbm24	-1.3927522	2.625791	down
Ripply1	-1.3905624	2.6218088	down
Nrap	-1.3900499	2.6208775	down
Mthfd2	-1.3811941	2.6048388	down
Tas1r1	-1.3744576	2.592704	down
Smyd1	-1.3737316	2.5914	down
Cox7a1	-1.373658	2.5912676	down
Klhl41	-1.3735566	2.5910854	down
Hspb7	-1.3728337	2.5897875	down
Chst13	-1.3721961	2.588643	down
Akr1c14	-1.3713768	2.5871735	down
Hephl1	-1.3709366	2.5863843	down

Myoz2	-1.3657546	2.5771108	down
Pdlim3	-1.3630309	2.5722501	down
Serpinb7	-1.355067	2.55809	down
Vgll2	-1.3514285	2.5516465	down
Opn3	-1.3424739	2.535858	down
Trim72	-1.3390884	2.5299141	down
Rdh5	-1.3380651	2.5281203	down
Gm7325	-1.3322892	2.5180192	down
Tmod1	-1.3297427	2.5135784	down
Pgm5	-1.3279359	2.5104325	down
Idi1	-1.3276005	2.5098488	down
Inpp4b	-1.3199956	2.4966536	down
Tnnc2	-1.3162975	2.490262	down
Btbd17	-1.316112	2.4899418	down
Casz1	-1.3156649	2.4891703	down
Fzd9	-1.3155534	2.488978	down
Nuak1	-1.306819	2.4739544	down
Myh7	-1.2991877	2.4609027	down
Rrm2	-1.2987809	2.4602091	down
Scn4a	-1.2983238	2.4594295	down
Actc1	-1.2864475	2.4392667	down
Mllt11	-1.2812479	2.4304912	down
Cap2	-1.281184	2.4303834	down
Kank1	-1.2771432	2.423586	down
Psg28	-1.2770437	2.4234188	down
Tspan32	-1.2767894	2.4229918	down
Casq2	-1.2692995	2.410445	down
Cgref1	-1.2664802	2.405739	down
Arpp21	-1.2661824	2.4052427	down
Hhatl	-1.26521	2.403622	down
Trim54	-1.2592845	2.39377	down
Abra	-1.258105	2.3918138	down
Tubb3	-1.2564063	2.388999	down
Prkaa2	-1.2543447	2.3855877	down
Mybpc1	-1.2491295	2.3769796	down
Nav2	-1.2425838	2.3662193	down
Sbk2	-1.2349777	2.3537772	down
Baiap2l1	-1.2338057	2.3518658	down
Pacsin3	-1.2326837	2.3500373	down
Msln	-1.2271523	2.3410444	down
Arhgef6	-1.2259331	2.3390667	down
Cldn2	-1.2253082	2.338054	down
Dusp27	-1.2218256	2.3324168	down
Nsdhl	-1.2119002	2.3164253	down
Daam2	-1.2087808	2.311422	down
Usp13	-1.2082596	2.3105872	down
Acsl6	-1.2033832	2.3027906	down
Gys1	-1.2017326	2.3001575	down
Gpr56	-1.1977782	2.2938614	down
Psg26	-1.1974187	2.29329	down
Tuba1a	-1.1960516	2.2911177	down
Rnd1	-1.1951964	2.28976	down
Klhl40	-1.1932216	2.2866278	down
Gm14137	-1.1926615	2.2857404	down
Aldh1l2	-1.1895938	2.2808852	down
Acta1	-1.1883974	2.2789946	down
Jph1	-1.188062	2.2784646	down
Il34	-1.1853595	2.2742004	down

Sorbs2	-1.1801121	2.2659438	down
Aif1l	-1.1796384	2.2652	down
Tbx15	-1.1766462	2.2605069	down
Awat2	-1.1764888	2.26026	down
Slc7a11	-1.1742402	2.25674	down
Tnnt1	-1.1741037	2.2565265	down
Myh3	-1.1708193	2.2513952	down
Baalc	-1.168307	2.247478	down
Palm3	-1.1636465	2.2402294	down
Tead4	-1.1608355	2.2358687	down
Tmsb10	-1.1602106	2.2349005	down
4-Sep	-1.1582754	2.2319045	down
Myh6	-1.1541784	2.2255754	down
Rassf7	-1.1533675	2.224325	down
Fyn	-1.1501217	2.219326	down
Cyp2b13	-1.1496562	2.21861	down
Lzts1	-1.1492661	2.2180104	down
Tuba4a	-1.1483631	2.2166226	down
Fgfr3	-1.1471334	2.2147338	down
Ankrd32	-1.146416	2.2136328	down
Tubb6	-1.1327219	2.1927204	down
Slc22a4	-1.1301142	2.1887608	down
Odc1	-1.1274772	2.1847637	down
Limch1	-1.1271468	2.1842635	down
Ablim3	-1.1230419	2.1780572	down
Hfe2	-1.1192455	2.1723335	down
Best1	-1.1153866	2.1665306	down
Psat1	-1.10889	2.1567965	down
Porcn	-1.1021252	2.1467068	down
Sgcg	-1.0958478	2.1373866	down
Synpo2	-1.0918837	2.1315215	down
Npnt	-1.0907569	2.1298575	down
Tmem8c	-1.090333	2.1292317	down
Fgf13	-1.0868517	2.1241	down
Klhl31	-1.084419	2.1205213	down
Scn10a	-1.081804	2.1166813	down
Mid1ip1	-1.0797372	2.113651	down
Akap6	-1.0789876	2.1125531	down
Myom3	-1.0766048	2.109067	down
Deptor	-1.0760715	2.1082873	down
Pgam2	-1.0756292	2.1076412	down
Tm6sf1	-1.0668347	2.0948322	down
Cdsn	-1.0645726	2.09155	down
Hrc	-1.0640993	2.0908642	down
Zbtb7c	-1.0624486	2.088473	down
Pet117	-1.0617347	2.08744	down
Chac1	-1.0558448	2.0789351	down
Cryab	-1.0551672	2.077959	down
Cdkn1a	-1.0538807	2.0761068	down
Mss51	-1.0533797	2.075386	down
Fdps	-1.0525942	2.0742564	down
Mfsd2a	-1.051641	2.0728862	down
Srpk3	-1.0498104	2.0702577	down
Dusp8	-1.047793	2.067365	down
Smim22	-1.0463862	2.06535	down
Sh3rf1	-1.0424042	2.059657	down
Adh1	-1.041979	2.05905	down
Klhl30	-1.0394034	2.0553775	down

Gdnf	-1.0384567	2.0540292	down
Kcnq4	-1.0357888	2.0502343	down
Hsf2bp	-1.0321009	2.045	down
Jsrp1	-1.0314093	2.04402	down
Gpm6b	-1.0313469	2.0439315	down
Epha4	-1.0287755	2.0402918	down
Bcap29	-1.0277231	2.038804	down
Ckb	-1.0252826	2.035358	down
Phgdh	-1.0245886	2.0343792	down
Ccdc141	-1.0240593	2.033633	down
Actn3	-1.0215597	2.0301125	down
Adssl1	-1.0206981	2.0289004	down
Ankrd23	-1.0194032	2.0270803	down
Tmem62	-1.0193355	2.0269852	down
Fam171a2	-1.0127945	2.0178158	down
Tnnt2	-1.0126514	2.0176158	down
Nsun3	-1.0110804	2.0154197	down
Rin1	-1.0099204	2.0138	down
Fndc5	-1.0072436	2.010067	down
Mylk4	-1.006602	2.0091734	down
Alpk3	-1.0056136	2.0077972	down
Gprc5c	-1.0052314	2.0072653	down
Cacnb1	-1.0047736	2.0066285	down
Mylk2	-1.0034748	2.004823	down
Amacr	-1.0028315	2.0039291	down
Herc3	-1.0027437	2.0038073	down
Nes	-1.0023718	2.0032907	down
Zfp61	1.0002203	2.0003054	up
Rnase4	1.0025427	2.003528	up
Ston1	1.0025699	2.0035658	up
Zscan29	1.002705	2.0037534	up
1700094D03Rik	1.0027355	2.0037959	up
Snrk	1.0029333	2.0040705	up
Gda	1.0039072	2.0054238	up
Clec2d	1.0044675	2.006203	up
Ifitm6	1.0046812	2.0065	up
Figf	1.0055113	2.007655	up
Slc36a1	1.0084629	2.0117667	up
Ralgds	1.010397	2.0144653	up
Tspyl2	1.011158	2.0155282	up
Raet1a	1.0116682	2.016241	up
Afap1l2	1.0129311	2.0180068	up
Gpr125	1.0136335	2.0189896	up
Zfp219	1.0139353	2.019412	up
1700017B05Rik	1.0160289	2.0223446	up
Ifitm3	1.0173564	2.0242064	up
Shc2	1.0177634	2.0247774	up
Mtmr11	1.0182825	2.0255063	up
Ackr3	1.0186338	2.0259995	up
Pnma1	1.0197588	2.02758	up
Spag1	1.0208867	2.0291657	up
Dpysl3	1.0213389	2.0298018	up
Rerg	1.0214512	2.02996	up
Rorc	1.0224081	2.0313067	up
Casp2	1.0225202	2.0314646	up
Efna1	1.0226436	2.0316384	up
Dpysl2	1.0237098	2.0331402	up
Naa16	1.0238469	2.0333335	up

Efna5	1.024467	2.0342076	up
Hs1bp3	1.0253167	2.035406	up
Tmem98	1.0260394	2.036426	up
Aebp1	1.0265155	2.0370982	up
Prdm15	1.0268345	2.0375485	up
Sema4c	1.0278974	2.0390503	up
Rab36	1.0311834	2.0437	up
Tekt2	1.0311834	2.0437	up
F3	1.0317535	2.0445077	up
Cbx6	1.0318627	2.0446625	up
Eya1	1.0324285	2.0454645	up
Mcam	1.0324286	2.0454648	up
Gfod2	1.0334417	2.0469015	up
Rgs2	1.0341913	2.0479653	up
Fscn1	1.0382156	2.0536861	up
Pltp	1.0385528	2.054166	up
Slc25a23	1.0386746	2.0543394	up
F11r	1.0393139	2.05525	up
Ccdc85c	1.0413795	2.0581946	up
Ralb	1.0427866	2.060203	up
Ripk1	1.0443096	2.0623791	up
Parp8	1.0453906	2.063925	up
Tgm2	1.0463352	2.0652769	up
Hexb	1.0468488	2.0660121	up
Doc2a	1.0473919	2.06679	up
Plaur	1.048651	2.0685947	up
Ccdc24	1.04964	2.0700133	up
Appl2	1.0499752	2.0704942	up
Ly6e	1.0501022	2.0706766	up
Ctns	1.0504305	2.071148	up
Gpr39	1.0506744	2.071498	up
Ctsb	1.0511603	2.0721958	up
Baiap2	1.0524008	2.0739784	up
Hoxc11	1.0527561	2.074489	up
Trp53inp1	1.0531192	2.0750113	up
Reep4	1.0542073	2.076577	up
Il18bp	1.0552359	2.078058	up
Blnk	1.0570486	2.0806706	up
Zbtb48	1.058041	2.0821023	up
Trip10	1.058794	2.0831895	up
Ddit3	1.0617886	2.087518	up
Rbm15b	1.0634105	2.0898662	up
Slc43a1	1.0653343	2.0926547	up
Plat	1.0655634	2.092987	up
Bdh2	1.0668267	2.0948205	up
Cfap69	1.0669118	2.0949442	up
Zscan22	1.0673423	2.0955694	up
Plxna3	1.0675087	2.0958111	up
Ticam1	1.0687921	2.0976763	up
Rnf24	1.0710976	2.1010313	up
Fjx1	1.0722119	2.1026545	up
Antxr2	1.0722713	2.1027412	up
Hes1	1.0723279	2.1028237	up
Bcl3	1.0735791	2.104648	up
Ctxn1	1.0745704	2.1060948	up
Gm2a	1.0761929	2.1084647	up
Man2b1	1.0764637	2.1088605	up
Prex2	1.0768763	2.1094637	up

Dgkq	1.0774838	2.1103523	up
Nid1	1.0795765	2.1134155	up
Filip1l	1.0797706	2.1137	up
P2rx6	1.0802696	2.1144311	up
Sh3gl3	1.0813601	2.11603	up
Zbp1	1.0822611	2.117352	up
Cbx2	1.0829902	2.1184223	up
Arl9	1.085037	2.12143	up
Lrch2	1.0861231	2.1230276	up
Mecom_dup1	1.0864102	2.12345	up
Capn15	1.0866171	2.1237547	up
Bag3	1.0870829	2.1244404	up
Atp6v1a	1.0874481	2.1249783	up
Fhod1	1.087687	2.1253302	up
Tm4sf1	1.0885081	2.1265402	up
Zmym1	1.0885565	2.1266115	up
4833427G06Rik	1.0901488	2.12896	up
Mov10	1.0939755	2.1346145	up
Sipa1	1.0958552	2.1373975	up
Rcan2	1.0981297	2.14077	up
Hsp90aa1	1.0982132	2.1408937	up
Cpne8	1.0996825	2.1430752	up
Fam102a	1.1043911	2.1500812	up
Bscl2	1.1046574	2.1504781	up
Irf1	1.1051707	2.1512434	up
Tmem173	1.1055663	2.1518333	up
Hoxa9	1.1086265	2.1564026	up
F2r	1.1099534	2.1583867	up
Zfp324	1.111986	2.16143	up
Prrg1	1.1129699	2.1629043	up
Plekho2	1.1132438	2.163315	up
Mtcp1	1.1136036	2.1638546	up
Slc25a27	1.1145337	2.16525	up
Znfx1	1.1190012	2.1719654	up
Slc27a3	1.119383	2.1725404	up
Grn	1.1212978	2.1754258	up
Ephb6	1.1235895	2.1788843	up
S1pr2	1.1241035	2.1796606	up
Sipa1l3	1.1250265	2.1810555	up
Snai2	1.1275561	2.184883	up
Fbxo46	1.127578	2.1849163	up
Six4	1.130733	2.1896996	up
Rassf5	1.1312602	2.1905	up
Hoxc13	1.1328986	2.1929889	up
Dbp	1.1343125	2.1951394	up
Anpep	1.1385629	2.201616	up
Wdr81	1.140562	2.204669	up
Igf2bp2	1.1423197	2.2073565	up
Ifi203	1.143017	2.2084239	up
Efemp1	1.1436106	2.2093325	up
Eml1	1.1438022	2.209626	up
Kirrel	1.1441784	2.2102022	up
BC022687	1.144278	2.210355	up
Frmd8	1.1444402	2.2106032	up
Ramp1	1.1482838	2.2165008	up
C1qtnf5	1.1489788	2.2175686	up
Phldb2	1.1547356	2.2264352	up
Cep95	1.1551661	2.2270997	up

Gnpda1	1.1553249	2.2273448	up
Rere	1.1564851	2.2291367	up
Elf1	1.1569846	2.2299085	up
Zbtb40	1.1584275	2.2321398	up
Plscr1	1.1585703	2.2323608	up
Sord	1.1600814	2.2347004	up
Mmp14	1.1614833	2.236873	up
Bcl2l11	1.1634673	2.2399511	up
Ifit3	1.1668503	2.24521	up
Zcchc2	1.1674576	2.2461553	up
Lasp1	1.1690054	2.2485662	up
Gins3	1.1727116	2.2543502	up
Hdac7	1.1727722	2.2544448	up
Tpbg	1.173995	2.2563565	up
Psrc1	1.1743331	2.2568853	up
Cthrc1	1.1760391	2.2595556	up
Spn	1.1782515	2.2630234	up
Rarg	1.1784186	2.2632856	up
Zfp53	1.1818521	2.2686784	up
Cpt1c	1.1829157	2.2703514	up
Swt1	1.1831697	2.2707512	up
Cadm1	1.1836505	2.2715082	up
Tmem140	1.1842804	2.2725003	up
Abcc5	1.1845427	2.2729132	up
Fdxr	1.1858156	2.2749195	up
Pcdhgb1	1.190053	2.2816112	up
Plekhg3	1.1904418	2.2822263	up
Dhrs3	1.1912562	2.2835147	up
Six5	1.1920704	2.2848039	up
Cmtm3	1.1932697	2.286704	up
Igfbp5	1.1932697	2.286704	up
Camkk1	1.1935687	2.287178	up
Twist1	1.19383	2.2875924	up
Epha1	1.1939061	2.287713	up
Vcam1	1.1950355	2.2895045	up
Pla2g7	1.1964775	2.2917943	up
Htra3	1.1966872	2.2921274	up
Mgp	1.1968641	2.2924085	up
Zfp36l2	1.2002053	2.2977238	up
Hoxa5	1.2051421	2.3056	up
Nsg1	1.2064645	2.3077142	up
Naglu	1.2106926	2.3144872	up
Lrrc45	1.2109635	2.3149219	up
Adamts10	1.2142849	2.3202574	up
Izumo4	1.2150178	2.3214364	up
Crim1	1.2153544	2.3219783	up
Ppbp	1.2162939	2.3234909	up
Mrc2	1.2174592	2.3253682	up
P2rx7	1.2176257	2.3256366	up
Gm14322	1.2178594	2.3260133	up
Mgme1	1.2181615	2.3265004	up
Gpr137b	1.2264328	2.3398771	up
Mtmr4	1.2276714	2.3418868	up
Rac3	1.229185	2.344345	up
Dll1	1.229372	2.344649	up
Enpp5	1.2321204	2.34912	up
Bex2	1.2334217	2.3512397	up
Lgals3bp	1.23389	2.3520033	up

Piga	1.235079	2.3539424	up
Capn6	1.2387369	2.359918	up
P4ha2	1.2394652	2.36111	up
Dzip1	1.2395822	2.3613014	up
Deaf1	1.2400422	2.3620543	up
Tcn2	1.243576	2.3678472	up
Smco4	1.2437239	2.36809	up
Slc25a37	1.2448506	2.3699403	up
9430015G10Rik	1.2507039	2.379575	up
Gfra1	1.255301	2.3871696	up
A230050P20Rik	1.2557086	2.387844	up
Klrg2	1.2623595	2.3988776	up
Postn	1.2632313	2.4003274	up
Dnaja1	1.2647309	2.402824	up
Fam212b	1.2674465	2.407351	up
Cfh	1.2754498	2.4207428	up
Gm20594	1.2756286	2.4210427	up
Tes	1.280623	2.4294386	up
Pwwp2b	1.2815542	2.4310074	up
Npff	1.2893039	2.444101	up
Arhgef39	1.2893107	2.4441125	up
Jag1	1.2904863	2.446105	up
Cxcl12	1.2936561	2.4514854	up
Pcdh10	1.2979515	2.458795	up
Soat1	1.3045335	2.4700384	up
Irf9	1.3053799	2.471488	up
Fam117a	1.305507	2.4717057	up
Crebl2	1.3088026	2.4773583	up
Efna4	1.3111027	2.4813113	up
Irf2bp2	1.3145337	2.4872193	up
Serping1	1.3153282	2.4885895	up
Trim46	1.3175263	2.492384	up
Dpp7	1.3228388	2.5015786	up
2310030G06Rik	1.3247176	2.5048387	up
Hoxc6	1.3252754	2.5058072	up
Grem1	1.3285592	2.5115173	up
Mrgprf	1.331259	2.5162218	up
Id3	1.3374305	2.5270085	up
Fam193b	1.3437948	2.5381808	up
Ndst4	1.3450172	2.5403323	up
Ebf3	1.3475692	2.5448298	up
Ifi27l2a	1.3524885	2.553522	up
Isg15	1.3537991	2.5558429	up
Lgals2	1.356425	2.5604992	up
Traf5	1.3610568	2.5687327	up
Tmc3	1.3647461	2.57531	up
Sprr1a	1.3669367	2.5792234	up
Ptprv	1.3733252	2.5906699	up
Sox4	1.3750267	2.593727	up
Mgat3	1.3776076	2.5983713	up
Eno2	1.3826096	2.607396	up
Snx33	1.3850064	2.6117313	up
Klk1b22	1.3888422	2.6186845	up
Rhou	1.3907753	2.6221957	up
Fos	1.3982977	2.6359036	up
Timp3	1.4011884	2.6411905	up
Dedd2	1.4015543	2.6418607	up
Angptl4	1.4079537	2.6536052	up

Car9	1.4148346	2.6662917	up
Itga10	1.419951	2.675764	up
Sap25	1.4211997	2.678081	up
Igflr1	1.4253747	2.6858425	up
Rgl1	1.4289324	2.6924741	up
Fam129c	1.4345455	2.70297	up
Asap3	1.4364549	2.7065496	up
Eps8	1.4447284	2.7221158	up
Twist2	1.446501	2.7254624	up
Socs3	1.4486456	2.7295167	up
Tnfsf9	1.4499942	2.7320695	up
Fam131b	1.4563222	2.7440794	up
Apol9a	1.4582831	2.7478116	up
Plau	1.4662066	2.7629445	up
Ikbke	1.4722843	2.7746086	up
Ifit1	1.4964112	2.8214	up
Ttll3	1.5044153	2.8370967	up
Plekhf1	1.5089512	2.8460307	up
Osr2	1.5175709	2.8630857	up
Mmd	1.5326905	2.893249	up
Ppp2r2b	1.5389888	2.9059076	up
Gpnmb	1.5495925	2.9273443	up
Cxcl1	1.562613	2.9538836	up
Cp	1.5715762	2.972293	up
Gpx3	1.5749991	2.979353	up
Atp6v0d2	1.5786344	2.9868698	up
Rhbdl1	1.5839695	2.9979358	up
Cfp	1.592174	3.0150335	up
Irs2	1.6122823	3.057351	up
Plscr2	1.6145887	3.062243	up
Cpe	1.6318688	3.099142	up
Adora2b	1.6345987	3.1050117	up
Prl2c2	1.6518807	3.1424303	up
Tspan7	1.6570457	3.1537006	up
Col11a2	1.6741807	3.1913807	up
Pgf	1.7268362	3.3100114	up
Plin2	1.7625742	3.39303	up
Serpine2	1.7733958	3.4185767	up
Ccl2	1.8030243	3.4895096	up
Efnb2	1.8506379	3.6065962	up
1810011O10Rik	1.8651463	3.6430488	up
Ccl5	1.8890802	3.70399	up
Ch25h	1.9376318	3.830763	up
Ccl8	1.9650378	3.9042294	up
Htr1b	2.0586944	4.166091	up
Dnajb1	2.1423616	4.4148417	up
Banp	2.194024	4.5758004	up
Hsph1	2.2049065	4.6104465	up
Kbtbd11	2.3435433	5.0754766	up
Plac8	2.4982524	5.650006	up
Slc5a3	3.3002431	9.850816	up
Hspa1b	3.9858406	15.843735	up

Supplementary Table S2. Primers for the expression analysis by RT-qPCR of the mRNAs indicated.

for mouse

Gene	Sequence	Product
Rpl13a	5’-GTGGTCCCTGCTGCTCTCAAG-3’	151bp
	5’-CGATAGTGCATCTTGGCCTTTT-3’	
Pax7	5’-AGGCCTTCGAGAGGACCCAC-3’	95bp
	5’-CTGAACCAGACCTGGACGCG-3’	
MyoD	5’-AGCACTACAGTGGCGACTCA-3’	75bp
	5’-GGCCGCTGTAATCCATCAT-3’	
Myog	5’-CAACCAGGAGGAGCGCGATCTCCG-3’	86bp
	5’-GGCGCTGTGGGAGTTGCATTCACT-3’	
Mymk	5’-GTATACTCCGGTCCCATAGGC-3’	81bp
	5’-CAGGCCCTTCTTCTCTTTCA-3’	
Myh1	5’-TCTGCAGACGGAGTCAGGT-3’	94bp
	5’-TTGAGTGAATGCCTGTTTGC-3’	
Myh3	5’-TCCAAACCGTCTCTGCACTGTT-3’	84bp
	5’-AGCGTACAAAGTGTGGGTGTGT-3’	
Myh7	5’-ATGCTGACAGATCGGGAGAA -3’	181bp
	5’-GGTTGGCTTGGATGATTTGA-3’	
Ckm	5' -CAGCACAGACAGACACTCAGG-3'	84bp
	5' -GAACTTGTTGTGGGTGTTGC-3'	
Ccnd1	5’-AGTGCGTGCAGAAGGAGATT-3’	89bp
	5’-CTCTTCGCACTTCTGCTCCT-3’	
Cdkn1a (p21)	5’-CCTCGGATCTCTGGTCAAGT-3’	107bp
	5’-GCAGGTACATGCTGATCATCTC-3’	
Cdkn2a (p16)	5’-ATGTCAGATGGGAACCCAGA-3’	74bp
	5’-GTCTTTGGGGTGGTTGGAG-3’	
Cdkn1b (p27)	5' -GTGGACCAAATGCCTGACTC- 3'	122bp
	5' -TCTGTTCTGTTGGCCCTTTT- 3'	
Tceal1	5' -GTACGAAACAAACTGATGATAATGC- 3'	74bp
	5' -AGTGGAGCATATTAAATAGGGTAAGG- 3'	
Tceal3	5' -GCAGAACCAGCTCCCTGA- 3'	116bp
	5' -GCTGGGAACAGCAGAGACC- 3'	
Tceal5	5' -GACTTGAAACTGAACGACAGAGG- 3'	94bp
	5' -GTTTCCTTGGCTGGGTTG- 3'	
Tceal6	5' -CACCGTTTCCTTGAGCTGTC- 3'	101bp
	5' -GTCACAATGCACTGGGAACA- 3'	
Tceal7	5' -ACTTGTGGCCAAGGAGAAGA- 3'	181bp
	5' -GGGTTGTTCATCCTCCCTCT- 3'	
Tceal8	5' -GAGTGGCCCAGCTACTTCC- 3'	107bp
	5' -GATTTGCAGGGGCCTTCT- 3'	
Tceal9	5' -TGTTCTTTGTACCGCACCAG- 3'	106bp
	5' -GGCAGGGTTTCATCTTGTGT- 3'	
